# The Effect of Cognitive-Behavioral Therapy on Acromegalics After a 9-Month Follow-Up

**DOI:** 10.3389/fendo.2019.00380

**Published:** 2019-06-12

**Authors:** Lia Silvia Kunzler, Luciana Ansaneli Naves, Luiz Augusto Casulari

**Affiliations:** ^1^Directory of Health, University of Brasilia, Brasilia, Brazil; ^2^Department of Endocrinology, Faculty of Medicine, University of Brasilia, Brasilia, Brazil; ^3^Endocrinology Service of the Brasilia University Hospital, Brasilia, Brazil; ^4^Clinic of Neurology and Endocrinology, Brasilia, Brazil

**Keywords:** acromegaly, cognitive-behavioral therapy, quality of life, follow-up, SF−36

## Abstract

**Introduction:** The quality of life of acromegalics is compromised. Treatment with the “Think healthy and feel the difference” technique has been described as effective in the improvement of the quality of life of these patients in the short term. However, its effectiveness in the long term needs to be evaluated.

**Objective:** The purpose of this study was to evaluate whether the good results obtained from the “Think healthy and feel the difference” technique in the short term persists in the long term, after the end of the treatment.

**Method:** This is a non-randomized longitudinal study with 23 acromegalic divided into two groups: an intervention group with 10 patients and a control group with 13 patients. The intervention itself covered nine group sessions, organized weekly, using a technique called “Think healthy and feel the difference.” The control group did not receive the aforementioned treatment. The Short Form 36 Question Health Survey (SF-36) and the Beck Depression Inventory were administered before the sessions began, at the end of the nine therapy sessions and at the 9-month follow-up.

**Results:** At the end of the treatment, the results of the SF-36 showed improvement in the mental health of the intervention group compared to the control group. The effects of therapy were maintained at the 9 month follow-up.

**Conclusion:** Cognitive-behavioral therapy, applied in a group format, can improve the quality of life of acromegalic patients in the short- and long-term.

## Introduction

In acromegaly, prolonged exposure to growth hormone (GH) and insulin-like growth factor (IGF-1) is associated with worsening comorbidities and impairment in quality of life (1). Biochemical dysregulation of the disease can reduce acromegalic life expectancy by up to 10 years (1, 2), but, when the disease is controlled, mortality may reach parity with the normal population (2). Despite this, biochemical control of acromegaly through currently available treatments—transsphenoidal surgery, radiotherapy and pharmacotherapy—has been insufficient to improve quality of life (2–4).

In addition to clinical complications, orofacial changes and orthopedic comorbidities can also impair the quality of both functional and social life (1). Acromegalics show a preoccupation with the performance of tasks involved in executive dysfunction syndrome, which may impair the execution of everyday tasks (5), as well as jeopardize the development of effective coping strategies (6). Receptors for GH and IGF-1 are present in the central nervous system, especially in the limbic system and frontal lobe. An excess of GH and IGF-1 results in cognitive impairment may be irreversible, as in valvulopathy and arthropathy (6).

Delay in diagnosis is related to social isolation, distortion of body image, and depression, with consequent psychosocial impairment (1, 7). The impairment in quality of life may also be related to psychiatric comorbidities, such as anxiety, cognitive impairment, and depression, more frequently than in other somatic diseases (1, 8).

Therefore, quality of life impairment may be related to psychopathology and not only to biochemical dysregulation of the disease (3). Cognitive dysfunctions due to emotional imbalance have been observed in acromegalics, especially in executive functions and in memory (5, 9, 10). Lower activation of areas of the brain strongly associated with cognitive functions—the prefrontal and temporal cortex—has been observed as well (5).

Considering the importance of psychopathology in acromegaly (3), particularly, related to the supposed irreversible effect of excess GH on mood and behavior (1), acromegalics can benefit from psychotherapeutic treatment (2, 8, 9). However, no results on the effects of psychotherapy on improvement of quality of life in acromegalics had been published until recently, despite being well-established for other chronic diseases (4).

In this context, cognitive-behavioral therapy (CBT) can be applied to cases of acromegaly. Over the years, research involving CBT has contributed to clinical practice, since improvements in techniques favor its application in different clinical contexts (11). More than 300 research studies have applied CBT to various diseases, as it is an effective intervention with a low cost (12).

Initially, the cognitive model, proposed by Aaron Beck in the 1960s, was based on the cognitive distortions present in depression (13). Depressive content was characterized by low self-esteem and guilt with a consequent desire for isolation (14). In CBT, thought, emotion, and behavior are intertwined (14, 15).

The cognitive model has been adapted for various diseases. In this model, schemas are sets of beliefs associated with greater emotional imbalance (13, 16, 17). Due to facial changes and growth of their hands and feet, (1), acromegalics can present schemas of inadequacy, devaluation and failure. As with acromegalics, depressed patients overestimate their defects, failures, and problems, and tend to underestimate or ignore any favorable traits, skills, or achievements (14).

The cognitive conceptualization of each case is facilitated by Socratic questioning. The therapist formulates questions to help the patient understand the effects of emotions and thoughts on behavior. Such questioning aims at developing new coping perspectives from the learning process (18).

Among several clinical applications, in social phobia, for example, the patient feels threatened in social situations, thinking “*If I try to talk, I'll look stupid”*; as a result the patient avoids social interaction (13). In acromegaly, because of their physical appearance, the cognitive model may be similar, based on the belief: “*If I look horrible, everyone will stare and criticize me.”*

Uncertainty, cost and benefit assessment, and perceived risk precede the choice of a given behavior, and require some degree of flexibility (19). Despite the importance of decision making, not much research has been carried out the influence of emotional imbalance on choices (20). In the brain, activation of the cingulate cortex and the amygdala is important because of their role in emotional regulation, which is one of the main objectives of CBT (21, 22).

In this context, the “Think healthy” technique was applied in a group setting with the aim of improving the quality of life of acromegalics (23). The technique is composed of six stages and uses the figures of gray and green avocados to represent problems and possible coping alternatives (23). One of its fundamental principles is decision making toward healthy behaviors, facilitated by the following thought: “*Despite the negative repercussions of acromegaly, what can I do that is healthier toward thinking and behavior*?”

In a previously published study, we showed that CBT, with the “Think Healthy” technique, significantly improved the quality of life of acromegalics; evaluated by the SF-36 in the areas of general health (d′ = −0.264, *p* = 0.031) and mental health (d′ = −1.123, *p* = 0.012). In the dimensions of physical and emotional aspects there were no significant improvement, although four of the five patients with floor effects did improve (23).

The aim of the present study is to evaluate whether the initially obtained results were still present after 9 months from the end of the treatment. It also compares whether, after that period of time, the treatment group shows any difference to the control group that did not receive the treatment at the same time.

## Methods

This was a non-randomized longitudinal study performed on acromegalics attending the neuroendocrinology outpatient clinic of the University Hospital of Brasília in the period from September 2015 to November 2016.

The following inclusion criteria were used: patients with acromegaly in follow-up at the above-mentioned clinic, males and females, aged between 18 and 75 years; intellectual capacity to follow the intervention; and agreement to participate in the study. The exclusion criterion was risk of suicide, evaluated according to the response to item 9 of the BDI (24).

The meetings were held in a space that comfortably accommodated the number of participants, arranged in a circle, with the possibility of handling the material, taking notes and completing exercises. The room was well lit, cool, and had good privacy.

The clinical trial was registered in a public trial registry, and the accession number is UTN—U 1111-1220-9846. The research project was approved by the Research Ethics Committee (CEP) of the Faculty of Health Sciences of the University of Brasília—UnB. A Free and Informed Consent form was read and signed by the participants of the group.

### Sample Composition and Evaluation Steps

One week before the start of group therapy, which was considered the pre-intervention stage, the work proposal, the dates scheduled for the meetings, and the content scheduled for each session were provided. Information was collected about age, gender, time, and activity of the disease, arterial hypertension, diabetes, arthralgia, and type of acromegaly treatment—surgery, radiotherapy and/or oral or injectable medication.

The disease was considered active when IGF-1 was above the normal value for age and gender, even when using cabergoline, octreotide, or pegvisomant; considered controlled when the use of these drugs kept blood concentrations of IGF-1 within the normal range; and considered cured when the concentrations of this hormone were within the normal range without the use of these drugs. Hypertensive participants were considered as those with blood pressure equal to or >140 × 90 mmHg, or those using anti-hypertensive drugs (25). A participant was considered to have diabetes mellitus type 2 when glycemia was equal to or higher than 125 mg/dL, or 2 h post oral glucose tolerance test higher than 199 mg/dL or HbA1 levels equal to or higher 6.5%, or if he/she was using hypoglycemic drugs (26).

The groups were selected according to convenience, availability, and the choice of each participant. Patients of the control group did not participate on this phase of the research due to the distance of their homes or difficulties with transportation. Despite the interest in participating, they did not manage to do so due to the need to be at the ambulatory on a weekly basis. Those who chose to participate in the therapy sessions were considered as the intervention group (IG)—aged between 42 and 69 years—and those who preferred not to participate in this phase of the experiment were considered as the control group (CG)—aged between 30 and 75 years. All participants in the two groups responded to the evaluation instruments before the treatment started.

The details of all steps of the treatment were clarified. Acromegalics attended weekly, one and a half hour group sessions that applied the technique “Think healthy and feel the difference” (23, 27–30); for a total of 9 weeks. The IG was treated in the 3 month period from September to November 2015.

In the post-intervention step, after the end of the nine sessions of application of the technique, all IG and CG participants were submitted to evaluations with the aim of analyzing the effect of CBT on the IG. The results were compared between groups.

**Figure 1:**
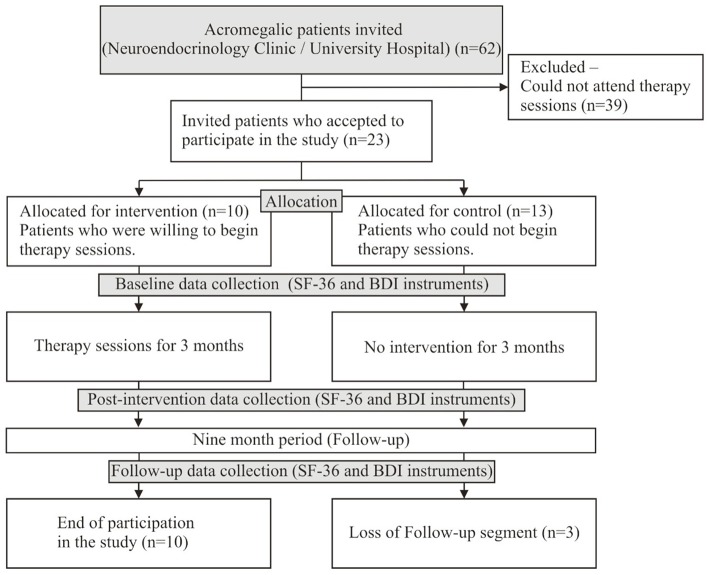
Sample composition and evaluation steps.

For the follow-up phase, in August 2016, 9 months after the end of the IG sessions, the IG and CG participants were once again submitted to evaluations. Three participants in the control group did not respond to the evaluation instruments. As a result, the total number of participants dropped from 23 to 20 (Figure 1).

### Evaluation Instruments

For acromegaly, AcroQol is the instrument used in 80% of studies to evaluate quality of life. It consists of 22 questions, 11 of them more directly related to physical alterations. Regarding the level of cognition, seven of the questions are conditional beliefs with emotional dysregulation of moderate intensity, as for example, item 8: “I feel rejected by people because of my illness.” Four of them are core beliefs with greater emotional dysregulation, for example, item 4: “I look awful in photographs.” Because of the risk of intense imbalance emotion, AcroQol questions were used only in the seventh session for the participants to identify their cognitive distortions, restructure them and build healthier behaviors.

Because of this, two self-administered evaluation instruments were answered by the participants of the two groups in the three stages of the present study: pre-intervention, post-intervention, and follow-up.

### Quality of Life Questionnaire (SF-36)

The Short Form 36 Question Health Survey (SF-36) was used to evaluate quality of life (31). This is a multidimensional questionnaire, composed of 36 items, subdivided into eight sections with their respective questions: physical functional (item 03); role-physical (item 04); bodily pain (items 07 and 08); general health (items 01 and 11); vitality (options a, e, g, and i of item 09); social functioning (items 06 and 10); role-emotional (item 05), and mental health (options b, c, d, f, and h of item 09). In addition to the eight sections, item 02 is not part of the calculation of any section, being used only to evaluate how much the individual is better or worse compared to a year ago. It presents a final score of 0 (zero) to 100 (hundred), in which zero shows the worst state and 100 the best state (31).

### Beck Depression Inventory (BDI)

The Beck Depression Inventory (BDI) was used to evaluate signs of depression (24). The following levels were considered for this scale, as they are for psychiatric patients: minimal: 0 to 11; mild: 12 to 19; moderate: 20 to 35; serious: 36 to 63. A score of 18 to 19 indicates likelihood of depression (24).

### Intervention Protocol

The “Think healthy and feel the difference” protocol (23, 27–30), adapted for patients with acromegaly, was applied in a group setting and all participants received printed material. The technique is available to download as an application for mobile devices at the *Apple Store and Google Play*, under the name “*Think Healthy*,” with versions in Portuguese and English. Patients allowed the researcher to record the sessions for later transcription.

A small, plastic, coping card, measuring 10 × 6 cm, with an illustration of the avocados, was delivered to encourage the practice of the techniques and healthy decision making in moments of emotional imbalance.

In each session, specific topics were covered. The content of the sessions was presented as follows: (1) basic concepts of CBT and practical examples of the relationship between unhealthy and healthy levels of cognition with emotion and associated behaviors; (2) emotional regulation; (3) increase self-esteem and self-confidence, and supply of a brain-shaped piggy bank (identification and valuation of personal qualities and achievements); (4) “Think healthy and feel the difference” technique—guidelines for completing the six steps adapted for the practice of physical activity; (5) the technique for approaching anger, assertiveness training, and the coping card; (6) the technique for approaching fear; (7) the technique adapted for shame about one's physical appearance in acromegaly; (8) clarifications on acromegaly, and (9) a summary of the topics covered in the sessions.

To consolidate knowledge and to assist in the practice of cognitive and behavioral skills between the therapy sessions, a therapy notepad and block of coping cards were provided (29).

### Data Analysis

To verify the equivalence of profiles of both the control and the intervention groups, we performed the non-parametric Mann-Whitney U test for the scalar variables (32). For the categorical variables, Chi-square or Fisher's tests were performed (32).

To control the impact of non-randomization on the distribution in the groups, we performed covariance analysis (ANCOVA) to compare the SF-36 and BDI measurements in the post intervention period. The baseline means were used as covariates; thus, the groups were equalized by their baseline in the post-intervention comparison (33, 34).

In order to verify whether the clinical effect was sustained 9 months after the intervention, the same procedure to compare the measures post-intervention was done; however, to it was added the measures of the post-intervention as a covariant, as well as the baseline. Moreover, the comparison between the intervention and the control groups was made. Student's *t*-Test, paired, was used when comparing the measures of the baseline and of the post-intervention, and whether the clinical effect was sustained.

To interpret the size of the effects, Cohen's criterion was adopted: small (d′ < 0.3), moderate (0.3 ≤ d′ < 0.5), or large (d′ ≥ 0.5) (35). In all analyses, statistical significance was considered to be *p* < 0.05. In order to determine if the sample size was adequate for the effect size, thus avoiding a type II error, *post-hoc* analysis was made using the G^*^POWER 3.1 software, where the acceptable statistical power found was β = 0.80 (36, 37).

## Results

Sixty-two acromegalics were selected from the endocrinology clinic of the University Hospital of Brasília. Thirty-nine of them did not agree to participate in the study, mainly because they could not attend the study site on a weekly basis. Thus, the sample consisted of 23 patients. There were 15 women (65.2%). Ages ranged from 30 to 75 years (mean = 52.78 ± 12.86 years, CV = 0.24, n = 23), from 42 to 69 years in the intervention group, and from 30 to 75 years in the control group. The mean time since disease diagnosis was 11.60 ± 7.14 years (CV = 0.62, n = 23). The time lag between the diagnosis and the beginning of the treatment ranged from 0.17 to 18 years (mean = 6.37 ± 4.94 years, CV = 0.78, n = 23). Regarding the stages of the disease, four (17.40%) were classified as cured, in 10 it was under control (43.50%), and in nine it was active (39.10%). The MRI assessment of the tumor showed the mean of the largest diameter was 23.36 ± 16.49 mm (CV = 0.71; n = 22). The percentage of IGF-1 levels (ULNR IGF-1) ranged from 30.30 to 384.30 (mean = 144.33 ± 85.00, CV = 0.59, n = 23). As for the treatment strategies, 20 (87%) underwent surgery, 21 (91.3%) used medication, and seven (30.40%) underwent radiotherapy. Regarding the level of depression, 11 (47.8%) presented minimal scores according to the BDI, six (26.1%) had mild scores, and six (26.1%) had moderate scores.

As shown on Table 1, there was no significant difference between all the variables studied: age, time since diagnosis, delay in diagnosis, largest tumor diameter in MRI, IGF-1 ULNR %, gender, disease activity, underwent therapies, and symptoms of depression. Among the variables in the study, only bodily pain presented significant differences (*p* = 0.002), where the control group had a higher score (mean = 66.67 ± 24.00) compared to the intervention one (mean = 38.89 ± 12.00).

**Table 1:** Comparison between the control group and the intervention group in relation to age, gender, the clinical and laboratory aspects of the disease, and the study variables in the baseline (*n* = 23).

	**Control (*n* = 13)**	**Intervention (*n* = 10)**	p-value^*^
	**Median (Q1–Q3)**	**Median (Q1–Q3)**	
Age (years)	55.00 (50.0–62.0)	46.00 (37.8–58.3)	0.335
Time since diagnosis (years)	11.00 (4.8–19.3)	12.00 (8.75–16.5)	0.926
Delay in diagnosis (months)	48.00 (24.0–108.0)	84.00 (66.0–120.0)	0.532
Largest diameter in MRI (mm)	20.00 (17.0–27.0)	12.00 (11.3–49.5)	0.815
ULNR IGF-1 (%)	117.45 (109.8- 121.3)	118.70 (103.38–245.88)	0.664
	**f(%)**	**f(%)**	***p*****-value^†^**
**Gender**			
Male	6 (46.15)	1 (10.00)	0.074
Female	7 (53.85)	9 (90.10)	
**Disease activity**			
Active	6 (46.15)	3 (30.00)	0.734
Controlled	5 (38.46)	5 (5.00)	
Cured	2 (15.38)	2 (2.00)	
**Underwent surgery**			
Yes	12 (92.31)	8 (80.00)	0.560
No	1 (7.69)	2 (20.00)	
**Using acromegaly medication**			
Yes	11 (84.62)	10 (100.00)	0.486
No	2 (15.38)	0 (0.0)	
**Underwent radiotherapy**			
Yes	3 (23.08)	4 (40.00)	0.650
No	10 (76.92)	6 (60.00)	
**Symptoms of depression (BDI)**			
Minimal	7 (53.85)	4 (40.00)	0.805
Mild	3 (23.08)	3 (30.00)	
Moderate	3 (23.08)	3 (30.00)

**Table:** 

**Study variables**	**Mean**	**Standard deviation**	**Mean**	**Standard deviation**	***p*****-value^‡^**
Physical functional	70.00	26.06	58.50	26.67	0.311
Role-physical	50.00	36.80	32.50	39.18	0.284
Bodily pain	66.67	24.00	38.89	12.00	0.002
General health	53.08	15.07	46.00	21.58	0.364
Vitality	58.85	14.74	55.00	10.00	0.487
Social functioning	62.50	27.95	67.50	14.67	0.614
Role-emotional	56.41	45.92	43.33	49.81	0.521
Mental health	67.08	20.73	62.00	15.23	0.523
Beck depression inventory	12.23	9.57	13.30	9.03	0.788

Q1, 1° Quartile; Q3, 3° Quartile;

* Non-parametric Mann-Whitney test;

† Chi-squared test;

‡ *t-test for repeated measurements*.

The results of the effect of the treatment on the intervention group compared to the control one (patients who did not receive treatment) are presented in Table 2. Concerning the SF-36 sections, Vitality was higher in the control group (mean = 66.15 ± 9.61; *p* = 0.041; β > 0.80). However, Mental Health (mean = 76.80 ± 11.12) was higher in the intervention group when compared to the control group (mean = 68.92 ± 15.68; *p* = 0.025; β > 0.80). There were no significant differences between both groups in all the other sections. Regarding the BDI, no significant differences were observed between the groups either.

**Table 2:** Effect of the treatment on the intervention group compared to the control group (no treatment) in relation to the quality of life questionnaire SF-36 and the Beck Depression Inventory (*n* = 23).

	**Control–post (*****n*** **=** **13)**			**Intervention–post (*****n*** **=** **10)**			***p*-value^**§**^**
	**Mean**	**Standard deviation**	***d^**′**^* post × pre**	**Mean**	**Standard deviation**	***d'* post × pre**	
Physical functional	66.92	26.34	−0.117	52.50	27.21	−0.223	0.481
Role-physical	59.62	47.37	0.228	65.00	42.82	0.793	0.252
Bodily pain	65.81	25.44	−0.035	50.00	17.57	0.752	0.705
General health	57.69	18.44	0.275*^†^*	51.50	20.15	0.264^†^	0.984
Vitality	66.15	9.61	0.600*^†^*	54.50	14.03	−0.042	0.041
Social functioning	77.88	17.04	0.684	76.25	18.11	0.534	0.571
Role-emotional	53.85	48.19	−0.155	76.67	35.31	−0.243	0.740
Mental health	68.92	15.68	0.101	76.80	11.12	1.123^*^	0.025
Beck depression inventory	7.54	7.69	−0.544	12.50	8.49	−0.091	0.139

d′- d′ of Cohen;

* *t-test for repeated measurements p<0.01*;

† *t-test for repeated measurements p < 0.05*;

§ *ANCOVA; Post: Intervention x Control, Covariant: Baseline (SF36 and BDI)*.

When comparing the baseline to the post-intervention, there was a significant increase in the score of General Health both in the control group (D′ = 0.280; *P* = 0.038; B > 0.80) and in the intervention one (D′ = 0.26; *P* = 0.024). Regarding the Vitality section, there was a significant increase only in the control group (D′ = 0.60; *P* = 0.022; β > 0.80). The score for Mental Health has a significant increase (*P* = 0.008), the greatest effect found (D′ = 1.123; β > 0.80). There were no significant differences in the other sections nor in the BDI evaluation.

Table 3 presents the adjustments measures for the ANCOVA models of the effect of the treatment in the intervention group compared to the control group, which did not receive the treatment, in the post-intervention. Concerning the SF-36, the sections were well-adjusted to the model, except for the Vitality variable (*R*^2^ = 0.176).

**Table 3:** Adjustment measures for the ANCOVA models (*n* = 23) when comparing the control group (no treatment) to the intervention group in the post-intervention in relation to the quality of life questionnaire SF-36 and the Beck Depression Inventory.

	**Control (*n* = 13)**	**Intervention (*n* = 10)**	***R*^2^**	**Base level standardization**
Physical functional	62.88	57.75	0.647	65.00
Role-physical	53.89	72.44	0.334^*^	42.39
Bodily pain	57.37	60.98	0.339^*^	54.59
General health	55.00	54.94	0.639^*^	50.00
Vitality	65.81	54.95	0.176	57.17
Social functioning	78.77	75.10	0.225^*^	64.67
Role-emotional	50.99	80.38	0.292^*^	50.72
Mental health	67.77	78.29	0.477^*^	64.87
Beck depression inventory	7.76	12.21	0.311^*^	12.70

R^2^, Model adjustment;

* *p < 0.001*.

Nine months after the end of the intervention, patients of both groups were again evaluated using SF-36 and BDI. Only the Mental Health section sustained a significant difference (*p* = 0.033) when comparing the control group (mean = 65.60 ± 15.91) to the intervention group (mean = 74.80 ± 21.25), in which case the intervention one had higher score (β > 0.80). The patients during the 9 months before the final evaluation had no changes in their disease stage.

The comparison between the post-intervention and the 9 month follow-up is presented in Table 4. The *t*-test for repeated measurements did not show significant difference either in the control group or the intervention group in any of the SF-36 sections or BDI.

**Table 4:** Comparisons of follow up between the control and intervention groups in relation to the quality of life questionnaire SF-36 and the Beck Depression Inventory (*n* = 20).

	**Control–follow-up (*****n*** **=** **10)**			**Intervention–follow-up (*****n*** **=** **10)**			*p*-value^*^
	**Mean**	**Standard deviation**	***d′***	**Mean**	**Standard deviation**	***d′***	
			**follow-up x post**			**follow-up x post**	
Physical functional	69.50	24.43	0.041	50.00	27.59	−0.091	0.364
Role-physical	58.33	43.30	0.130	50.00	45.64	0.413	0.989
Bodily pain	56.67	20.59	−0.448	51.11	24.68	0.053	0.852
General health	54.00	16.47	−0.215	47.00	20.58	−0.221	0.255
Vitality	59.50	12.12	−0.562	50.00	14.14	−0.319	0.395
Social functioning	73.75	18.11	−0.296	65.00	27.51	−0.493	0.308
Role–emotional	55.56	47.14	−0.155	53.33	50.18	−0.546	0.970
Mental health	65.60	15.91	−0.133	74.80	21.25	−0.124	0.033
Beck depression inventory	11.40	12.02	0.424	10.60	6.24	−0.258	0.465

d′- d′ of Cohen;

* ANCOVA, Post: Intervention x Control, Covariant: Baseline and Post (SF36 and BDI)

Table 5 presents the adjustment measures for the ANCOVA models when comparing the follow-up between the groups. Regarding the SF-36 instrument, only the Bodily Pain section (*R*^2^ = 0.156), General Health Perception (*R*^2^ = 0.141) and Mental Health (R^2^ = 0.351) were well-adjusted to the model.

**Table 5:** Adjustment measures for the ANCOVA models (*n* = 20) when comparing the control group (no treatment) and the intervention one, during the follow-up, in relation to the quality of life questionnaire SF-36 and the Beck Depression Inventory.

	**Control (*n* = 10)**	**Intervention (*n* = 10)**	***R*^2^**	**Base level standardization**	
				**Baseline**	**Post**
Physical Functional	64.215	55.285	0.488	63.75	60.50
Role-Physical	53.792	54.087	0.018	42.11	65.79
Bodily Pain	55.152	52.626	0.156^*^	52.22	58.33
General Health	55.645	45.355	0.141^*^	51.00	54.75
Vitality	57.384	52.116	0.221	57.25	60.25
Social Functioning	75.03	63.72	0.017	63.75	77.50
Role-Emotional	53.908	54.816	0.059	52.63	70.13
Mental Health	60.997	79.403	0.351^*^	63.60	72.20
Beck Depression Inventory	12.53	9.47	0.149	12.50	9.80

R^2^, Model adjustment;

* *p <0.01*.

## Discussion

The results showed that the application of the “Think Healthy” technique, for 9 weeks, resulted in an improvement in the quality of life in the intervention group when compared to the control group. This confirms the previous observation that the technique is useful in psychological support of acromegalics. (23). Furthermore, the present study supplements that the effect was maintained at a 9 month follow-up, which suggests stability in the effects produced by the treatment.

The strategy used here—nine sessions of group therapy—should be effective, since in groups with obsessive-compulsive disorder, a program of seven sessions was as effective as the same program conducted over 12 sessions (38).

Thus, the good long-term results, described here, of the use of CBT in improving quality of life in acromegalics fills a gap in the management of these patients. This is because, the psychotherapeutic approach has been described as important in other chronic diseases, but not in acromegaly (4).

Regarding the results achieved in the SF-36, in the intervention group, the mental health dimension had a significant increase, showing the greatest effect with the treatment (Table 4). In both groups there was an improvement in general health, which could be explained by the fact that all participants of the study where treated on the same neuroendocrinology outpatient clinic of the University Hospital of Brasília.

In the control group, the difference in the vitality variable should be interpreted with caution, since it did not present a good fit for the model (*R*^2^ = 0.176) (Table 3). This result shows the need of having other studies as well to try and explain this finding. What could have happened is that the IG participants have learned to observe themselves while the CG participants stayed lenient to their evaluation. The other dimensions did not show significant differences, although some presented moderate and large effects, and fitted the model well.

The improvement in quality of life after treatment and the fact that it was maintained after a 9 month follow-up may have been facilitated by the characteristics of CBT. The intervention stimulates visual memory, because it uses visual, verbal, and written support. Tasks based on CBT require planning and execution—executive function—and can be considered cognitive training. Socratic questioning—characteristic of the systematization of the technique in its six stages—favors the learning and active participation of patients in their treatment process (18).

In terms of outcome and maintenance of the achieved improvement, the results of this research are similar to those obtained in other studies on other diseases. Improvements achieved in obsessive-compulsive symptoms were maintained in evaluations after a 1-year (38) and a 2-year follow-up (39). Positive results were also achieved and maintained in follow-ups for other disorders: anxiety in young people (40), obesity with binge eating (41), general anxiety, (42) psychiatric disorders in comorbidity (43), compulsive shopping (44), social anxiety (17), and body dysmorphic disorder in adolescents (45).

The results described here, using CBT to improve quality of life, are of particular importance because it is still uncertain whether the control of the disease is associated with some gain in this parameter. For example, with biochemical control of acromegaly, a study with a longer duration of follow-up (mean follow-up 5.7 ± 0.6 years) did not show improvement in quality of life when compared to the control group (46). These authors emphasized that addressing physical and psycho-social comorbidities is as important as the biochemical control of the disease (46). This study (45) evaluated the quality of life of 106 surgically treated patients, 3 and 12 months after the surgical intervention. Regarding the SF-36, there was improvement in all dimensions, but it was not statistically significant (47).

On the other hand, a study that used the SF-36 to evaluate the quality of life of 41 acromegalics before surgery, after surgery, and at a 1-year follow-up, showed a statistically significant improvement in the following dimensions: role-physical, general health, social functioning, and mental health (7). These results were similar to those described in this study for the domains of general health and mental health.

The short-lasting treatment and the possibility of recurrence at the end of the treatment are of real concern; however, in the present study the results were maintained at a 9-month follow-up. Some participants reported the benefits of the group format for learning coping strategies. This may be because learning and living experiences, such as psychotherapy, can induce epigenetic changes in brain circuits, similar to the effect of medication (48). When pharmacology is combined with psychotherapy, due to therapeutic synergy, the result tends to be greater than the sum of the two treatments (1 + 1 = 3) (48).

In the present study, the BDI score levels of the psychiatric patients (i.e., minimum, mild, moderate, and severe) were used because of the lack of a cutoff point for patients with acromegaly. In the pre-intervention assessment, three participants showed an indication of moderate depression, three showed mild depression, and four showed minimal depression. No participant in the present study showed an indication of severe depression. The lowest depression score possible (i.e., zero) was found in only one study participant. In that case, no improvement was possible, which is considered a floor effect. In addition, the decrease in BDI score was not significant.

Even though there is no gender difference between both groups, there was a tendency, which may point to a limitation in the generalization of the study. For this reason, the authors suggest that new gender stratification studies be carried in the future.

A limitation of the present study is the small case-by-case analysis; however, acromegaly is a rare disease. In addition, we used a convenience sample, in which the patients presented for the treatment, rather than a random sample. However, the psychotherapeutic approach was showed to reduce the suffering and improve the quality of life for acromegalics, with good short- and long-term results. The results achieved by this study reinforce the importance of more research on the use of cognitive-behavioral techniques in different clinical contexts.

## Ethics Statement

The research project was approved by the Research Ethics Committee (CEP) of the Faculty of Health Sciences of the University of Brasília—UnB. A Free and Informed Consent form was read and signed by the participants of the group.

## Author Contributions

LK, LC, and LN analyzed the patient data. LK and LC were major contributions in writing the manuscript. LK is a cognitive-behavioral therapist by The Beck Institute and conducted the cognitive-behavioral group sessions. All authors agreed to be accountable for the content of the work and approved the submitted version of the manuscript.

### Conflict of Interest Statement

The authors declare that the research was conducted in the absence of any commercial or financial relationships that could be construed as a potential conflict of interest.

## Abbreviations

BDI: beck depression inventory
CBT: cognitive-behavioral therapy
CG: control group
GH: growth hormone
IG: intervention group
IGF: insulin-like growth factor-1
MRI: magnetic resonance imaging
SF-36: quality of life questionnaire.

## References

[1] Abreu A Tovar AP Castellanos R Valenzuela A Giraldo CMG Pinedo AC. Challenges in the diagnosis and management of acromegaly: a focus on comorbidities. Pituitary. (2016) 19:448–57. 10.1007/s11102-016-0725-2 27279011 PMC4935749

[2] Siegel S Streetz-van der Werf C Schott JS Nolte K Karges W Kreitschmann-Andermahr I. Diagnostic delay is associated with psychosocial impairment in acromegaly. Pituitary. (2013) 16:507–14. 10.1007/s11102-012-0447-z 23179964

[3] Geraedts VJ Dimopoulou C Auer M Schopohl J Stalla GK Sievers C. Health outcomes in acromegaly: depression and anxiety are promising targets for improving reduced quality of life. Front Endocrinol. (2015) 5:229. 10.3389/fendo.2014.00229 25610427 PMC4285111

[4] Geraedts VJ Andela CD Stalla GK Pereira AM van Furth WR Sievers C. Predictors of quality of life in acromegaly: no consensus on biochemical parameters. Front Endocrinol. (2017) 8:40. 10.3389/fendo.2017.00040 28316591 PMC5334635

[5] Shan S Fang L Huang J Chan RCK Jia G Wan W. Evidence of dysexecutive syndrome in patients with acromegaly. Pituitary. (2017) 20:661–7. 10.1007/s11102-017-0831-9 28823099

[6] Pereira AM Tiemensma J Romijn JA Biermasz NR. Cognitive impairment and psychopathology in patients with pituitary diseases. Netherlands J Med. (2012) 70:255–60. 10.1159/000314317 22859416

[7] Fujio S Arimura H Hirano H Habu M Bohara M Moinuddin FM. Changes in quality of life in patients with acromegaly after surgical remission - A prospective study using SF-36 questionnaire. Endocrine J. (2017) 64:27–38. 10.1507/endocrj.EJ16-0182 27681883

[8] Szcześniak D Jawiarczyk-Przybyłowska A Rymaszewska J. The quality of life and psychological, social and cognitive functioning of patients with acromegaly. Adv Clin Exp Med. (2015) 24:167–72. 10.17219/acem/3926 25923102

[9] Crespo I Santos A Valassi E Pires P Webb SM Resmini E. Impaired decision making and delayed memory are related with anxiety and depressive symptoms in acromegaly. Endocrine. (2015) 50:756–63. 10.1007/s12020-015-0634-6 26018738

[10] Leon-Carrion J Martin-Rodriguez J F Madrazo-Atutxa Soto-Moreno A Venegas-Moreno E Torres-Vela E. Evidence of cognitive and neurophysiological impairment in patients with untreated naive acromegaly. J Clin Endoc Metab. (2010) 95:4367–79. 10.1210/jc.2010-0394 20554710

[11] David D Cristea I Hofmann SG. Why cognitive behavioral therapy is the current gold standard of psychotherapy. Front Psychiatry. (2018) 9:4. 10.3389/fpsyt.2018.00004 29434552 PMC5797481

[12] Hofmann SG Asnaani A Vonk IJJ Sawyer AT Fang A. The efficacy of cognitive behavioral therapy: a review of meta-analyses. Cognit Ther Res. (2012) 36:427–40. 10.1007/s10608-012-9476-1 23459093 PMC3584580

[13] Beck AT Haigh EAP. Advances in cognitive theory and therapy: the generic cognitive model. Annu Rev Clin Psychol. (2014) 10:1–24. 10.1146/annurev-clinpsy-032813-153734 24387236

[14] Beck AT. Thinking and depression - I - Idiosyncratic content and cognitive distortions. Arch Gen Psychiatry. (1963) 9:324–33. 10.1001/archpsyc.1963.01720160014002 14045261

[15] Beck AT Knapp P. Cognitive therapy: foundations, conceptual models applications and research. Rev Bras Psiquiatr. (2008) 30(Suppl. 2):S54–64. 10.1590/S1516-44462008000600000210.1001/archpsyc.1964.01720240015003 19039445

[16] Beck AT. Thinking and depression - II – Theory and therapy. Arch Gen Psychiatry. (1964) 10:561–71. 10.1001/archpsyc.1964.01720240015003 14159256

[17] de Oliveira IR Powell VB Wenzel A Caldas M Seixas C Almeida C Efficacy of the trial-based thought record, a new cognitive therapy strategy designed to change core beliefs, in social phobia. J Clin Phar Ther. (2012) 37:328–34. 10.1111/j.1365-2710.2011.01299.x 21955037

[18] Braun JD Strunk DR Sasso KE Cooper AA. Therapist use of Socratic questioning predicts session-to-session symptom change in cognitive therapy for depression. Behav Res Ther. (2015) 70:32–7. 10.1016/j.brat.2015.05.004 25965026 PMC4449800

[19] Fellows LK. The cognitive neuroscience of human decision making: a review and conceptual framework. Behav Cogn Neurosci Rev. (2004) 3 (30) 159–72. 10.1177/1534582304273251 15653813

[20] Bechara A The role of emotion in decision-making: evidence from neurological patients with orbital damage. Brain Cogn. (2004) 55:30–40. 10.1016/j.bandc.2003.04.001 15134841

[21] Beck AT. The evolution of the cognitive model of depression and its neurobiological correlates. Am J Psychiatry. (2008) 165:969–77. 10.1176/appi.ajp.2008.08050721 18628348

[22] Klumpp H Fitzgerald JM Kinney KL Kennedy AE Shankman SA Langenecker SA. Predicting cognitive behavioral therapy response in social anxiety disorder with anterior cingulate cortex and amygdala during emotion regulation. Neuroimage Clin. (2017) 15:25–34. 10.1016/j.nicl.2017.04.006 28462086 PMC5403806

[23] Kunzler LS Naves LA Casulari LA. Cognitive-behavioral therapy improves the quality of life of patients with acromegaly. Pituitary. (2018) 21:323–33. 10.1007/s11102-018-0887-1 29644512

[24] Cunha JA Manual da versão em Português das Escalas Beck. São Paulo: Casa do Psicólogo (2001).

[25] Gomes MAM Nobre F Amoedo C Kohlmann O Praxedes JN Machado CA IV diretrizes brasileiras de hipertensão arterial. Arq Bras Cardiol. (2004) 82(Suppl. 4):7–22. 10.1590/S0066-782X2004001000004 15322661

[26] The Expert Committee on the Diagnosis and Classification of Diabetes Mellitus Follow-up report on the diagnosis of diabetes mellitus. Diabetes Care. (2003) 26:3160–7. 10.2337/diacare.26.11.3160 14578255

[27] Ferreira de Lima MCGP Kunzler LS Terapia cognitivo-comportamental nas doenças reumáticas. Capital Reum. (2017) 2017:14–9.

[28] Kunzler LS A contribuição da terapia cognitiva para o tratamento do transtorno obsessive-compulsivo. Brasília Med. (2008) 45:30–40.

[29] Kunzler LS Terapia cognitiva: aplicabilidade da técnica Pense saudável. Rev Debates Psiquiatr. (2015) 5:28–37.

[30] Kunzler LS Araujo TCCF. Cognitive therapy: using a specific technique to improve quality of life and health. Estudos Psicol. (2013) 30:267–74. 10.1590/S0103-166X2013000200013

[31] Ciconelli RM Ferraz MB Santos W Meinão I Quaresma MR Tradução para a língua portuguesa e validação do questionário genérico de avaliação de qualidade de vida SF-36 (Brasil SF-36). Rev Bras Reumatol. (1999) 39:143–50.

[32] Gibbons J Chakraborti S Nonparametric Statistical Inference. 5th ed. New York, NY: Taylor & Francis (2011).

[33] Hair J Black W Babin B Rolph A Tatham R Análise Multivariada de Dados. Porto Alegre: Bookman (2005).

[34] Tabachnik BG Fidell LS Using Multivariate Analysis. Need Heights, MA: Allyn and Bacon (2001).

[35] Cohen J. The statistical power of abnormal-social psychological research: a review. J Abnormal Soc Psychol. (1962) 65:145–53. 10.1037/h0045186 13880271

[36] Faul F Erdfelder E Lang AG Buchner A. G^*^Power 3: a flexible statistical power analysis program for the social, behavioral, and biomedical sciences. Behav Res Methods. (2007) 39:175–91. 10.3758/BF03193146 17695343

[37] Faul F Erdfelder E Buchner A Lang AG. (2009). Statistical power analyses using G^*^Power 3.1: tests for correlation and regression analyses. Behav Res Methods. (2009) 41:1149–60. 10.3758/BRM.41.4.1149 19897823

[38] Himle JA Rassi S Haghigthatgou H Krone KP Nesse RM Abelson J. Group behavioral therapy of obsessive-compulsive disorder: seven- vs. twelve-week outcomes. Depress Anxiety. (2001) 13:161–5. 10.1002/da.1032 11413562

[39] Braga DT Manfro GG Niederauer K Cordioli AV. Full remission and relapse of obsessive-compulsive symptoms after cognitive-behavioral group therapy: a two-year follow-up. Rev Bras Psiquiatr. (2010) 32:164–8. 10.1590/S1516-44462010000200012 20658055

[40] Kendall PC Southam-Gerow MA. Long-term follow-up of a cognitive-behavioral therapy for anxiety-disordered youth. J Consult Clin Psychol. (1996) 64:724–30. 10.1037/0022-006X.64.4.724 8803362

[41] Agras WS Telch CF Arnow B Eldredge K Marnell M. One-year follow-up of cognitive-behavioral therapy for obese individuals with binge eating disorder. J Consult Clin Psychol. (1997) 65:343–7. 10.1037/0022-006x.65.2.343 9086701

[42] Dugas MJ Ladouceur R Léger E Freeston MH Langlois F Provencher MD. Group cognitive-behavioral therapy for generalized anxiety disorder: treatment outcome and long-term follow-up. J Consult Clin Psychol. (2003) 71:821–5. 10.1037/0022-006X.71.4.821 12924687

[43] Hagen R Nordahl HM Kristiansen L Morken G A randomized trial of cognitive group therapy vs. waiting list for patients with co-morbid psychiatric disorders: effect of cognitive group therapy after treatment and six and twelve months follow-up. Behav Cogn Psychother. (2005) 33:33–44. 10.1017/S1352465804001754

[44] Mitchell JE Burgard M Faber R Crosby RD de Zwaan M. Cognitive behavioral therapy for compulsive buying disorder. Behav Res Ther. (2006) 44:1859–65. 10.1016/j.brat.2005.12.009 16460670

[45] Krebs G de la Cruz LF Monzani B Bowyer L Anson M Cadman J. Long-term outcomes of cognitive-behavioral therapy for adolescent body dysmorphic disorder. Behav Ther. (2017) 48:462–73. 10.1016/j.beth.2017.01.001 28577583

[46] Kyriakakis N Lynch J Gilbey SG Webb SM. Impaired quality of life in patients with treated acromegaly despite long-term biochemically stable disease: results from a 5-years prospective study. Clin Endocrinol. (2017) 86:806–15. 10.1111/cen.13331 28316090

[47] Milian M Honegger J Gerlach C Psaras T. Health-related quality of life and psychiatric symptoms improve effectively within a short time in patients surgically treated for pituitary tumors - a longitudinal study of 106 patients. Acta Neurochir. (2013) 155:1637–45. 10.1007/s00701-013-1809-7 23836354

[48] Stahl SM. Psychotherapy as an epigenetic ‘drug’: psychiatric therapeutics target symptoms linked to malfunctioning brain circuits with psychotherapy as well as with drugs. J Clin Pharm Ther. (2012) 37:249–53. 10.1111/j.1365-2710.2011.01301.x 22594604

